# Effects of Community Nursing Simulation Education on Nursing Core Competencies, Clinical Judgment, and Clinical Performance of Nursing College Students

**DOI:** 10.3390/ejihpe15060092

**Published:** 2025-05-23

**Authors:** Hoo-Jeung Cho, Kyong-Sun Chong

**Affiliations:** Department of Nursing, Kyungnam College of Information & Technology, 45 Jurye-ro, Sasang-gu, Busan 47011, Republic of Korea; sunck71@hanmail.net

**Keywords:** patient simulation, community health nursing, competency-based education

## Abstract

This study aimed to evaluate the effects of community nursing simulation education on the nursing core competencies, clinical performance, and clinical judgment in home-visit nursing of nursing college students. A nonequivalent control group pretest–post-test design was used. Data were collected in August 2024 from the control group (*n* = 65) and in February 2025 from the experimental group (*n* = 64), with both groups comprising fourth-year students older than 20 years and from the same nursing college in Korea. Data analysis included an independent *t*-test carried out using SPSS 25.0 software. We found significant differences between the control and experimental groups in terms of the students’ nursing core competence (*t* = 4.88, *p* < 0.001, Cohen’s *d* = 0.86), clinical judgment (*t* = 4.53, *p* < 0.001, Cohen’s *d* = 0.80), and clinical competence (*t* = 4.52, *p* < 0.001, Cohen’s *d* = 0.00). The simulation education program applied in this study can be utilized as an intervention for nursing college students and be further developed for nursing students from other universities.

## 1. Introduction

### 1.1. Research Necessity

Clinical practicum education is a core component of nursing education, bridging theoretical learning and practice. In Korea, it plays a crucial role in transitioning students into competent professional nurses with practical skills (Ko & Kim, 2019). This is because clinical practicum allows students to apply theoretical knowledge in real-life situations and acquire the skills needed in healthcare settings. However, owing to the rapidly evolving healthcare system and advances in clinical practice, there is a shortage of resources and infrastructure for clinical training. Some hospitals have implemented alternatives, such as remote or vacation training, due to the high number of practicum students (Shin et al., 2017). Additionally, many nursing students report experiencing fear of direct interaction with patients and stress related to making mistakes (Yu, 2015). The barriers to effective clinical learning include communication gaps with healthcare professionals and educators, refusal to engage in care activities due to enhanced patient rights, and a lack of knowledge regarding advanced medical equipment and its operation (Shin et al., 2017). These factors ultimately compromise the quality of the clinical practicum.

In community health nursing, clinical practicums are conducted in public health centers and industrial sites. The number of nursing graduates in South Korea increased by 10.1 per 100,000 people, from 32.3 in 2015 to 42.4 in 2020, surpassing the OECD average of 31.4 (Ministry of Health and Welfare, 2022). The increasing demand for additional practicum institutions presents significant challenges in providing adequate practicum environments at public health centers and their branches, particularly given the rising number of students pursuing these opportunities. Consequently, some students must complete practicums in distant areas. Further, owing to the shift toward patient-centered care services, the scope of nursing activities that students can perform is very limited, leading to minimal direct care experience (Chin et al., 2018). Moreover, the underdeveloped educational systems of practicum institutions and the difficulty in securing qualified clinical instructors leave students feeling like unwelcome guests or surplus personnel (Chin et al., 2018), making effective community health nursing practicums challenging.

Simulation-based learning, increasingly recognized as an effective approach to supplement limited clinical training, is an innovative educational method that recreates realistic clinical situations to promote active learner engagement and provide hands-on experience in a safe environment (S. H. Kim et al., 2014). In nursing education, simulation-based training fosters critical thinking through facilitating discussions between learners and educators during debriefing sessions, improves communication and clinical performance skills, and enhances practicum satisfaction and confidence (M. N. Kim et al., 2016). The overall effectiveness of simulation-based education has been found to be significant, with particularly strong impacts in psychomotor and cognitive domains (S. H. Kim et al., 2014). These findings suggest that simulation-based training significantly contributes to improving nursing students’ practical skills and knowledge. Simulation-based learning is regarded as an effective educational method for adapting to the evolving healthcare landscape and the increasing complexity of nursing practice. It is expected to remain a vital part of nursing education and serve as a valuable supplement to clinical training (S. H. Kim et al., 2014; Song & Kim, 2023).

Community health nursing practicum plays a vital role in enhancing nursing students’ core competencies and clinical performance and judgment in home-visit nursing (Kwon et al., 2022). Nursing core competencies include professional attitudes, critical thinking, effective communication, and clinical performance. These skills are essential in clinical practice and serve as the foundation for nurses to effectively perform their duties in clinical settings (Kwon et al., 2022; J. M. Park et al., 2015). In relation to improving clinical performance and judgment in home-visit nursing, practicum education that includes simulation is effective in enhancing nursing students’ clinical capabilities. Through scenarios based on real patient situations, students can apply the nursing process using critical thinking, implement key nursing skills suited to the situation, and respond with appropriate nursing actions (J. S. Park et al., 2015). Moreover, a critical thinking disposition is a crucial factor in performing nursing tasks in clinical settings. It enables nurses to apply, analyze, synthesize, and infer information, as well as logically present their opinions and make sound decisions (Yang, 2019).

To date, research on simulation-based community health nursing practicum education has been limited. In Korea, a literature review published in March 2023 (Oh & Park, 2023) indicated that among 25 studies on simulation-based learning in nursing, none explicitly addressed community health nursing. Internationally, a systematic review (Aslan, 2021) investigated simulation practice in public health and community nursing education. Further, a study in Japan (Yoshioka-Maeda & Naruse, 2021) evaluated the effect of simulation-based health guidance in community settings on public health nursing students. Another study conducted in Israel (Dopelt et al., 2023) demonstrated the effectiveness of simulation training. Against this backdrop, the present study aims to investigate the effects of simulation-based community health nursing practicum education on nursing students’ nursing competencies and clinical performance and judgment in home-visit nursing. By doing do, this study seeks to provide foundational data to inform content aspects, such as scenario topics, algorithms, and the development of evaluation tools, and operational aspects, such as team composition and the assignment of roles necessary for problem-solving. This information may prove valuable for designing future simulation-based learning experiences.

### 1.2. Research Purpose

This study aims to examine the effects of community health nursing simulation-based education on nursing students’ nursing core competencies and clinical performance and judgment in home-visit nursing.

### 1.3. Research Hypotheses

Based on the literature review above, the following hypotheses are proposed:

**Hypothesis** **1.** *The experimental group that receives community health nursing simulation-based education will show greater improvement in nursing core competencies than the control group that does not receive the education.*

**Hypothesis** **2.** *The experimental group that receives community health nursing simulation-based education will show greater improvement in clinical judgment in home-visit nursing than the control group that does not receive the education.*

**Hypothesis** **3.** *The experimental group that receives community health nursing simulation-based education will show greater improvement in clinical performance in home-visit nursing than the control group that does not receive the education.*

## 2. Materials and Methods

### 2.1. Research Design

This study used a quasi-experimental nonequivalent control group pretest–post-test design (Table 1) to examine the effects of community health nursing simulation-based education on nursing students’ core nursing competencies and clinical performance and judgment in home-visit nursing. To prevent the diffusion of experimental effects, data were collected from 1–22 August 2024, for the control group and from 1–21 February 2025, for the experimental group.

### 2.2. Study Participants

Participants were selected from among fourth-year nursing students at K University in City B who understood the purpose of the study through a posted recruitment notice and voluntarily agreed to participate. The inclusion criteria included (1) adults aged 20 or older; (2) those who were enrolled as fourth-year students in the department of nursing at K University in City B at the time of the study; and (3) those who could read Korean and communicate without difficulty. In quasi-experimental studies, a direct relationship between the researcher and participants can introduce various biases, such as selection bias or conflict of interest, that may compromise the internal validity of the study (Schweizer et al., 2016). To mitigate this risk, students who were being directly advised by the researcher were excluded from this study.

The required sample size was calculated using the G*Power 3.1 program (Faul et al., 2009). After conducting two-sample and two-tailed *t*-tests, with an effect size (d) of 0.5, a significance level (α) of 0.05, and a power (1 − β) of 0.80, the minimum number of participants required per group was calculated to be 64.

**Figure 1:**
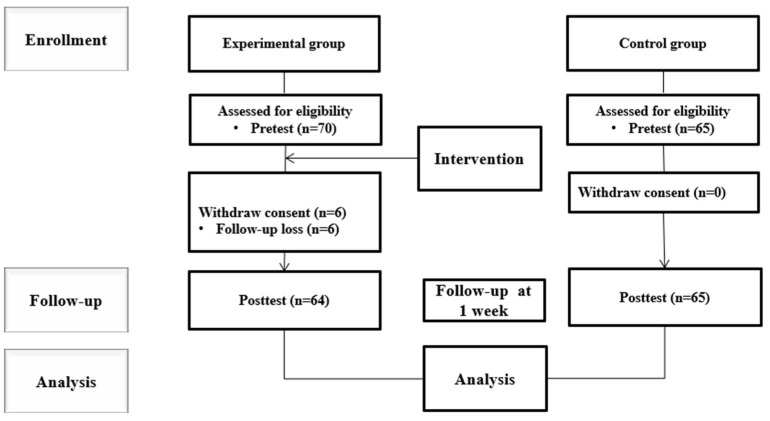
CONSORT of flow.

In the experimental group, among the fourth-year nursing students at K University in City B who participated in the simulation class from 1–21 February 2025, 70 met the inclusion criteria and completed the pretest. However, six students did not respond to the post-test survey, resulting in an attrition rate of 8.6%. The final number of participants in the experimental group was 64 (Figure 1).

In the control group, among the fourth-year nursing students at K University in City B who participated in the simulation class from 1–22 August 2024, 65 met the inclusion criteria and completed the pretest. There were no dropouts, and all the participants completed the post-test, resulting in a final control group of 65 students (Figure 1).

### 2.3. Research Instruments

#### 2.3.1. Nursing Core Competencies

Nursing core competencies were measured using a tool originally developed by Lu et al. (2023) for Chinese nurses and later revised and supplemented by Shi (2021). The tool comprises 40 items across 7 subdomains: critical thinking and research ability (5 items), clinical nursing skills (6 items), leadership (8 items), interpersonal skills (6 items), legal and ethical practice (5 items), professional development (4 items), and education and counseling (6 items). Each item is rated on a 5-point Likert scale from 1 (“strongly disagree”) to 5 (“strongly agree”), with higher scores indicating greater core nursing competency. The reliability of the tool showed a Cronbach’s α of 0.98 in Shi’s (2021) study and 0.97 in the present study.

#### 2.3.2. Clinical Judgment in Home-Visit Nursing

For this study, clinical judgment was measured using the Lasater Clinical Judgment Rubric (Lasater, 2007) to assess clinical judgment in simulation-based education, which was translated by Shim (2012). The tool includes 27 items, each rated on a 5-point Likert scale from 1 (“strongly disagree”) to 5 (“strongly agree”), with higher scores indicating stronger clinical judgment in home-visit nursing. The original tool had a Cronbach’s α of 0.88; Shim (2012) reported a reliability of 0.84, and the current study showed a Cronbach’s α of 0.89.

#### 2.3.3. Clinical Performance in Home-Visit Nursing

Clinical performance in home-visit nursing was assessed using a tool based on Schwarzer et al.’s (1997) self-reported six-dimension scale of nursing performance, which was revised by Choi (2005). The scale was further adapted for this study after content validation by two professors specializing in community health nursing with over 10 years of teaching experience and three public health center visiting nurses. A structured 4-point scale questionnaire was used to assess content validity, evaluated using the Content Validity Index for Items (I-CVI). Each item was rated as “highly appropriate” (4 points), “appropriate” (3 points), “inappropriate” (2 points), or “highly inappropriate” (1 point). The CVI for all the items was 1.00, indicating that all the experts deemed every item valid.

The tool comprises 20 items covering five areas of clinical performance. These include four items each for the nursing process, nursing skills, education and collaboration, interpersonal relations and communication, and professional development. Each item is scored on a 5-point Likert scale from 1 (“strongly disagree”) to 5 (“strongly agree”), with higher scores indicating stronger clinical performance in home-visit nursing. The original tool had a Cronbach’s α of 0.96; Choi (2005) reported a reliability of 0.92, and the current study showed a Cronbach’s α of 0.96.

### 2.4. Research Procedure

#### 2.4.1. Community Health Nursing Simulation-Based Education: Researcher Preparation

The co-investigator conducting the community health nursing simulation-based education in this study has over 10 years of clinical and teaching experience in community health nursing and possesses the professional expertise necessary to conduct the simulation-based training.

#### 2.4.2. Community Health Nursing Simulation-Based Education

The community health nursing simulation-based education applied in this study was developed to enhance the comprehensive nursing performance and problem-solving skills required in clinical practice among nursing students with prior clinical training experience. The learning module focused on “Nursing Care for Older Adults with Chronic Illnesses” and used standardized patients (SPs). The developed learning scenario was reviewed for structure and content validity by two professors in community health nursing with over 10 years of experience and five public health center visiting nurses. The CVI was measured and found to be 0.88.

To manage the simulation-based training and ensure quality, the design components for simulation instruction proposed by Watts et al. (2021) were incorporated. Following a simulation guide, the students completed pre-briefing in teams and participated in a 15–20 min simulation session using SPs. During the session, the students assessed nursing problems and carried out interventions based on their assigned roles and the scenario (Table 2). The SPs were professional actors who met face-to-face with the researcher beforehand to review and refine the scenario flow and scripts. The simulation-based training lasted approximately 1 h and 30 min.

During the simulation, the instructor monitored the students’ performance from the control room and could manipulate the situation if necessary. After each team completed their session, the students engaged in a 20 min debriefing session to receive feedback and evaluate the training. For efficient operation, each team consisted of four to five members.

#### 2.4.3. Pretest

During the pretest period (1–22 August 2024, for the control group and 1–21 February 2025, for the experimental group), the participants received an explanation of the study’s purpose and procedure and provided written informed consent. The participants were instructed on how to complete the questionnaires and asked to fill out items on general characteristics, core nursing competencies, and clinical performance and judgment in home-visit nursing. The average time required was 20 to 25 min.

#### 2.4.4. Implementation of Community Health Nursing Simulation-Based Education

The simulation-based education was implemented for approximately three days from 11–13 February 2025. The training was conducted directly by the co-investigator and lasted 80 min.

#### 2.4.5. Post-Test

For the control group, the post-test was conducted seven days after completing the pretest, between 1 August 2024 and 22 August 2024. For the experimental group, the post-test was conducted between 1 February 2025 and 21 February 2025, using the same questionnaires and procedures as the pretest. To reduce attrition, the questionnaires were collected immediately after completion and reviewed. The participants completed the questionnaires in a quiet, private space. The principal investigator distributed and collected the questionnaires, with the average time required being 20 to 25 min.

### 2.5. Ethical Considerations

This study was approved by the Public Institutional Review Board (https://public.irb.or.kr/) (format: assessed on 24 March 2025). To ensure voluntary participation, cooperation was requested from the school and department, and a recruitment notice was posted on the bulletin board in the nursing department office. Before the study began, all the participants received an explanation of the study’s purpose, procedures, anonymity, confidentiality, lack of disadvantages for participation, and that the collected data would only be used for research. Additionally, it was explained that participation was voluntary and could be withdrawn at any time. Informed consent was obtained before data collection. The participants were provided with contact information for inquiries, and the data were coded and stored in a secure file to prevent unauthorized access. The data will be stored for two years after the study and then destroyed. All the participants received a small token of appreciation for participating. After applying the intervention to the experimental group, the same simulation-based training was provided to the control group.

### 2.6. Data Analysis

The data collected in this study were analyzed using SPSS/WIN version 25.0 (IBM Co., Armonk, NY, USA), with a two-tailed test at the 5% significance level. First, the general characteristics and the homogeneity of the dependent variables between the experimental and control groups were analyzed using descriptive statistics and *t*-tests. Second, the normality of the pretest scores between the groups was tested using independent *t*-tests. Third, the effects of the intervention on core nursing competencies and clinical judgment and performance in home-visit nursing were analyzed using independent *t*-tests based on the differences between the pretest and post-test scores. Fourth, the effect sizes were calculated to minimize the impact of group size differences and to assess the actual magnitude of the differences between the groups. Finally, the internal consistency of the instruments used to measure the dependent variables was calculated using Cronbach’s alpha.

## 3. Results

### 3.1. General Characteristics of Participants and Homogeneity Test

The general characteristics of the study participants are as follows. In the control group, 10 participants (15.4%) were male and 55 (84.6%) were female; in the experimental group, 9 participants (14.1%) were male and 55 (85.9%) were female. The average age of the control group was 23.55 ± 3.87 years, while that of the experimental group was 24.72 ± 3.50 years. In terms of major satisfaction, the most common response in the control group was “satisfied” (35 participants, 53.8%), while in the experimental group it was “neutral” (30 participants, 46.9%). Regarding prior simulation-based education experience, 62 participants (95.4%) in the control group and 59 participants (92.2%) in the experimental group reported no experience. The most common grade point average (GPA) range was “3.5–3.9” in both groups, with 29 participants (44.6%) in the control group and 29 participants (45.3%) in the experimental group (Table 3). No significant differences were found between the two groups in terms of gender (*t* = 0.21, *p* = 0.834), age (*t* = 1.79, *p* = 0.076), major satisfaction (*t* = 1.83, *p* = 0.070), prior simulation experience (*t* = −0.75, *p* = 0.456), or GPA (*t* = −0.35, *p* = 0.724), indicating group homogeneity (Table 3).

### 3.2. Homogeneity Test of Pre-Intervention Dependent Variables

The results of the homogeneity test for pre-intervention dependent variables are shown in Table 4. Before the simulation-based education, core nursing competencies were 3.62 ± 0.60 and 3.58 ± 0.53 in the control and experimental groups, respectively (*t* = −0.37, *p* = 0.712); clinical judgment in home-visit nursing was 3.66 ± 0.42 and 3.55 ± 0.42 in the control and experimental groups, respectively (*t* = −1.47, *p* = 0.145). These showed no significant differences, indicating homogeneity. However, clinical performance in home-visit nursing was 3.78 ± 0.56 and 3.78 ± 0.59 in the control and experimental groups, respectively (*t* = 0.05, *p* = 0.005), showing a significant difference and thus a lack of homogeneity (Table 4).

### 3.3. Hypothesis Testing

#### 3.3.1. Hypothesis 1

To measure changes in nursing core competencies before and after the intervention, the difference in scores was analyzed using an independent *t*-test. The change was –0.12 ± 0.82 for the control group and 0.57 ± 0.79 for the experimental group, indicating a significant difference (*t* = 4.88, *p* < 0.001, Cohen’s *d* = 0.86), thus supporting Hypothesis 1 (Table 5).

#### 3.3.2. Hypothesis 2

To measure changes in clinical judgment in home-visit nursing before and after the intervention, the difference in scores was analyzed using an independent *t*-test. The change was –0.12 ± 0.78 for the control group and 0.53 ± 0.83 for the experimental group, indicating a significant difference (*t* = 4.52, *p* < 0.001, Cohen’s *d* = 0.80), thus supporting Hypothesis 2 (Table 5).

#### 3.3.3. Hypothesis 3

To measure changes in clinical performance in home-visit nursing before and after the intervention, the difference in scores was analyzed using an independent *t*-test. The change was –0.10 ± 0.54 for the control group and 0.42 ± 0.75 for the experimental group, indicating a significant difference (*t* = 4.53, *p* < 0.001, Cohen’s *d* = 0.00), thereby supporting Hypothesis 3 (Table 5).

## 4. Discussion

This study attempted to determine the effects of applying community health nursing simulation-based education on students’ core nursing competencies and clinical judgment and performance in home-visit nursing. To support the competencies required in clinical practice, this study applied simulation-based training using SPs in a setting that closely resembled a community health nursing environment and examined its effects.

After the community health nursing simulation-based training, the experimental group showed improved core nursing competencies compared to the control group, supporting Hypothesis 1. This result aligns with that of Kwon et al. (2022), who studied in-campus simulation-based education among 109 fourth-year nursing students. The specific core nursing competencies may vary depending on the clinical field; however, nursing competencies generally involve an integration of knowledge, including professional judgment, skills, values, and attitudes (Kwon et al., 2022), and are essential for nursing practice. In this study, core nursing competencies showed the most improvement. These competencies form the foundation of nursing practice and are essential for delivering high-quality healthcare services. Simulation-based education promotes clinical skill acquisition by mimicking real healthcare environments using high-fidelity scenarios (Sadeghi & Cohen, 2023). Prior studies have also found that nursing students who received scenario-based training showed statistically significant improvements in clinical skills compared to control groups, particularly in applying basic medical and scientific knowledge (Sadeghi & Cohen, 2023). Similarly, pediatric nursing simulation-based training improved nursing students’ critical thinking and problem-solving abilities (S. H. Kim et al., 2014), supporting the present findings. These findings suggest that teaching methods focused on real-world cases in settings that simulate clinical environments can enhance nursing students’ core competencies. Given the limitations on clinical practicums due to infectious diseases and pandemics like COVID-19 and the saturation of available training sites because of increasing student numbers, it is necessary to develop scenarios and diverse content focused on real-world cases, along with simulation-based training that incorporates appropriate teaching methods.

After the community health nursing simulation-based training, the experimental group showed improved clinical judgment in home visiting nursing compared to the control group, supporting Hypothesis 2. This is consistent with Lee’s (2017) study, which found that clinical judgment improved after simulation-based training among second-year nursing students with no prior simulation experience. This finding is also closely related to nursing competency. In community-based nursing simulation-based education, communication with clients is key, and for this, students’ confidence and problem-solving abilities are essential (Y. J. Kim & Park, 2016). According to Lim (2021), nursing process performance significantly improved after virtual reality simulation-based training, confirming its effectiveness in enhancing nursing process performance. These findings may directly relate to the complex judgment skills required in home-visit nursing settings. Confidence, problem-solving ability, and nursing process performance are essential for nursing students to assess complex home environments and make quick, accurate decisions as visiting nurses. Additionally, clinical judgment is especially crucial in home-visit nursing compared to other nursing fields, as home care environments are often unpredictable and typically lack immediate access to other healthcare professionals or resources. Nurses must independently assess patients, make timely decisions, and adapt care plans based on rapidly changing situations, all without direct supervision (Lim, 2021). The significant improvement in clinical judgment after simulation-based training appears to result from repeated experiences, including pre-briefing sessions where students evaluate patient situations and confirm identified problems, and debriefing sessions where they analyze and reflect on feedback regarding their behaviors and attitudes. Therefore, simulation-based training is an effective method for improving clinical judgment.

After the community health nursing simulation-based training, the experimental group showed improved clinical performance in home-visit nursing compared to the control group, supporting Hypothesis 3. Ahn and Lee (2021) reported that nursing students who received a home-visit virtual reality simulation program showed improved clinical performance compared to the control group. Kang and Kang (2020) also found that nursing students improved their clinical performance after receiving simulation-based education, supporting the results of this study. Simulation-based education provides students with the opportunity to practice in environments similar to real-world clinical settings, thereby enhancing their practical skills. Simulation realistically presents the challenges of leadership, decision-making, and teamwork while effectively highlighting the importance of meaningful and respectful discussion (Dopelt et al., 2023). Furthermore, by allowing students to apply theoretical knowledge and nursing skills in an integrated manner through repeated learning in a safe environment, simulation-based education proves to be an effective method for improving the clinical performance of nursing students. To further advance simulation-based education, it is necessary to develop scenarios that are not merely skill-focused but reflect complex and diverse situations that resemble real-life settings.

This study confirmed that community health nursing simulation-based education is an effective intervention for enhancing core nursing competencies, as well as clinical judgment and performance in home-visit nursing. From a clinical practice perspective, it is expected to support new nurses in acquiring practical skills on-site. From an educational standpoint, it offers efficient nursing education that helps narrow the gap between knowledge and practice for nursing students. From a research perspective, this study is significant in that it provides a foundation for the development of an educational program based on community health nursing.

Despite its contributions, this study has some limitations. First, this study was conducted using a convenience sample of fourth-year nursing students from K University in City B, which limits the generalizability of the findings. Second, since the participants were students, double blinding was not possible during data collection, and the researcher’s expectations of the intervention effect might have influenced the participants’ responses. Third, to prevent diffusion of the educational program, data collection for the experimental and control groups was conducted at different times. This time gap might have resulted in the influence of different extraneous variables between the two groups. Additionally, the pretest homogeneity test revealed a significant difference in clinical performance between the experimental and control groups, indicating a lack of homogeneity, which might have affected the study results.

## 5. Conclusions

This study aimed to examine the changes in core nursing competencies, as well as clinical judgment and performance in home-visit nursing before and after community health nursing simulation-based education among nursing students. The results show that simulation-based education improves core nursing competencies, as well as clinical judgment and performance in home-visit nursing among nursing students. The findings lay the groundwork for considering operational aspects in future simulation-based instructional design. This study is significant in that it verifies the effectiveness of simulation-based education based on community health nursing and emphasizes the importance of all three variables.

Based on these results, further research is needed across various dimensions by adjusting the level of simulation-based education according to learner variables, instructional design strategies, and implementation methods. Finally, studies using SPs should be conducted not only in the field of community health nursing but also across other nursing specialties.

## Figures and Tables

**Table 1:** Nonequivalent control group pretest–post-test design.

Group	Pretest	Intervention	Post-Test	Pretest	Intervention	Post-Test
Control group	C1 ^1^	X	C2 ^2^			
Experimental group				E1 ^1^	Xe ^3^	E2 ^2^

^1^ C1, E1: pretest (general characteristics, nursing focus competence, clinical judgment, and clinical competence); ^2^ C2, E2: post-test (nursing focus competence, clinical judgment, and clinical competence); ^3^ Xe: community nursing simulation education.

**Table 2:** Simulation theme, setting, and scenario; class goals; and tasks for students.

Theme	Class Goals	Setting	Scenario	Tasks for Students
Nursing care for older patients with chronic diseases	Assess and understand the patient’s health problems Perform health management services (health education, medication consultation) necessary for the patient Link the patient to community resource services Document nursing records for nursing activities performed	Standardized patient with elderly make-up (bruise makeup on right knee) Local simulation lab with home environment Visiting bag Blood glucose meter, blood pressure meter, thermometer, alcohol swab, hand sanitizer, nursing record	Mr. Jeong Ji-yeok (78 years old) lives alone in a semi-basement one-room apartment as a basic livelihood recipient. He has been taking medication for high blood pressure for the past two years, and during his last visit, his blood sugar level was measured at 180 mg/dL two hours after eating, and his glycated hemoglobin was 6.1%, requiring management. The patient is classified as belonging to the regular management group and receives visiting nursing services once every three months. You are a visiting nurse working at a public health center and provide visiting health management services.	Vital sign check Simple blood sugar check Assessment of patient Recording

**Table 3:** Homogeneity test for general characteristics of participants in experimental and control groups (*N* = 129).

Characteristics	Categories	Cont ^1^ (*n* = 65)	Exp ^2^ (*n* = 64)	*t*	*p*
		*N* (%)	*N* (%)		
Gender	Male	10 (15.4)	9 (14.1)	0.21	0.834
	Female	55 (84.6)	55 (85.9)		
Age (year)	Mean ± SD ^3^	23.55 ± 3.87	24.72 ± 3.50	1.79	0.076
Major satisfaction	Very dissatisfied	2 (3.1)	0 (0.0)	1.83	0.070
	Dissatisfied	3 (4.6)	4 (6.3)		
	Neutral	16 (24.6)	30 (46.9)		
	Satisfied	35 (53.8)	27 (42.2)		
	Very satisfied	9 (13.8)	3 (4.7)		
Simulation education experience (utilizing person)	Yes	3 (4.6)	5 (7.8)	−0.75	0.456
	No	62 (95.4)	59 (92.2)		
Grade point average	2.4 below	1 (1.5)	1 (1.6)	−0.35	0.724
	2.5–2.9	8 (12.3)	3 (4.7)		
	3.0–3.4	15 (23.1)	20 (31.3)		
	3.5–3.9	29 (44.6)	29 (45.3)		
	4.0–4.5	12 (18.5)	11 (17.2)		

^1^ Cont: control group; ^2^ Exp: experimental group; ^3^ SD: standard deviation.

**Table 4:** Homogeneity test of dependent variables (baseline) (*N* = 129).

Variables (Item Number)	Cont (*n* = 65)	Exp (*n* = 64)	*T*	*p*
	Mean ± SD	Mean ± SD		
Nursing focus competence (40)	3.62 ± 0.60	3.58 ± 0.53	−0.37	0.712
Clinical judgment (27)	3.66 ± 0.42	3.55 ± 0.42	−1.47	0.145
Clinical competence (20)	3.78 ± 0.56	3.78 ± 0.59	0.05	0.005

**Table 5:** Effectiveness of the education intervention program on nursing focus competence, clinical judgment, and clinical performance (*N* = 129).

Variables (Item Number)	Groups	Pretest	Post-Test	Differences (Post–Pre)	*t*	*p*
		Mean ± SD	Mean ± SD	Mean ± SD		
Nursing focus competence (40)	Exp	3.58 ± 0.53	4.15 ± 0.56	0.57 ± 0.79	4.88	<0.001
	Cont	3.62 ± 0.60	3.49 ± 0.55	−0.12 ± 0.82		
Clinical judgment (27)	Exp	3.55 ± 0.42	3.97 ± 0.54	0.42 ± 0.75	4.53	<0.001
	Cont	3.66 ± 0.42	3.55 ± 0.34	−0.10 ± 0.54		
Clinical competence (20)	Exp	3.78 ± 0.59	4.31 ± 0.51	0.53 ± 0.83	4.52	<0.001
	Cont	3.78 ± 0.56	3.66 ± 0.55	−0.12 ± 0.78		

## Data Availability

The original contributions presented in this study are included in the article. Further inquiries can be directed to the corresponding author(s).

## Abbreviations

The following abbreviations are used in this manuscript:

OECD	Organization for Economic Cooperation and Development
I-CVI	Content Validity Index for Items
SPs	Standardized patients
GPA	Grade point average
Exp	Experimental group
Cont	Control group
SD	Standard deviation
